# Therapy optimization in patients with heart failure: the role of the wearable cardioverter-defibrillator in a real-world setting

**DOI:** 10.1186/s12872-018-0790-8

**Published:** 2018-03-15

**Authors:** Susanne Röger, Stefanie L. Rosenkaimer, Anna Hohneck, Siegfried Lang, Ibrahim El-Battrawy, Boris Rudic, Erol Tülümen, Ksenija Stach, Jürgen Kuschyk, Ibrahim Akin, Martin Borggrefe

**Affiliations:** 1 First Department of Medicine, University Medical Centre Mannheim (UMM), Faculty of Medicine Mannheim, University of Heidelberg, Theodor-Kutzer-Ufer 1-3, 68167 Mannheim, Germany; 2 German Centre for Cardiovascular Research (DZHK), Partner Site Heidelberg/Mannheim, Mannheim, Germany

**Keywords:** Heart failure, Ischemic cardiomyopathy, Non-ischemic cardiomyopathy, Subcutaneous ICD, Sudden cardiac death, Wearable cardioverter-defibrillator

## Abstract

**Background:**

The wearable cardioverter-defibrillator (WCD) has emerged as a valuable tool to temporarily protect patients at risk for sudden cardiac death (SCD). The aim of this study was to determine the value of the WCD for therapy optimization of heart failure patients.

**Methods:**

One hundred five consecutive patients that received WCD between 4/2012 and 9/2016 were included in the study. All patients were followed for clinical outcome and echocardiographic parameters during WCD therapy and had continued follow-up after WCD therapy, irrespective of subsequent implantable cardioverter-defibrillator (ICD) implantation.

**Results:**

The most common indication for WCD were newly diagnosed ischemic (ICM) or non-ischemic cardiomyopathy (NICM) with left ventricular ejection fraction (LVEF) ≤35%. Mean WCD wear time was 68.8 ± 50.4 days with a mean daily use of 21.5 ± 3.5 h. Five patients (4.8%) received a total of five appropriate WCD shocks.

During WCD wear, patients with ICM and NICM showed significant improvement in LVEF, reducing the proportion of patients with a need for primary preventive ICD implantation to 54.8% (ICM) and 48.8% (NICM). An ICD was finally implanted in 51.4% of the study patients (24 trans-venous ICDs, 30 subcutaneous ICDs).

After discontinuation of WCD therapy, all patients were followed for a mean of 18.6 ± 12.3 months. 5.6% of patients with implanted ICDs received appropriate therapies. No patient with subcutaneous ICD needed change to a trans-venous device. None of the patients without an implanted ICD suffered from ventricular tachyarrhythmias and no patient died suddenly.

In patients with NICM a significant LVEF improvement was observed during long-term follow-up (from 34.8 ± 11.1% to 41.0 ± 10.2%).

**Conclusions:**

WCD therapy successfully bridged all patients to either LVEF recovery or ICD implantation. Following WCD, ICD implantation could be avoided in almost half of the patients. In selected patients, prolongation of WCD therapy beyond 3 months might further prevent unnecessary ICD implantation. The WCD as an external monitoring system contributed important information to optimize device selection in patients that needed ICD implantation.

## Background

Sudden cardiac death (SCD) causes about 13% of deaths in the overall population and about 36% of deaths in heart failure patients [1]. Clinical trials have shown the benefit of the implantable cardioverter-defibrillator (ICD) for treatment of SCD in both patients with ischemic (ICM) and non-ischemic cardiomyopathies (NICM) [2, 3].

Risk stratification for SCD is mainly based on left ventricular ejection fraction (LVEF) [4]. However, in patients with newly diagnosed heart failure, prior to medical therapy or in patients soon after myocardial infarction (MI) LVEF may improve with medical treatment obviating the indication for ICD. In the VALIANT trial patients with impaired LVEF were at highest risk for SCD in the first 30 days after MI [5]. Despite this risk, ICDs do not improve overall survival early after MI; lower rates of arrhythmic deaths appear counterbalanced by more non-arrhythmic deaths [6]. Current heart failure guidelines recommend optimal medical treatment (OMT) for at least 3 months before ICD implantation in both patients with ICM and NICM [4]. Recent data suggest that even longer waiting periods are appropriate in patients with newly diagnosed NICM [7]. This creates a window of susceptibility of several months when the propensity for malignant arrhythmias is high and no prophylactic therapy is provided.

The wearable cardioverter-defibrillator (WCD) provides a non-invasive temporary therapeutic option for patients during a period when the risk of SCD is changing or unclear.

Data from large registries have demonstrated safety and efficacy of WCD [8–10]. Episodes of sustained ventricular arrhythmias were recorded in about 1–3% of the patients during approximately 3 months of WCD use depending on WCD indication [8–10]. In the 2015 ESC Guidelines for the management of patients with ventricular arrhythmias and the prevention of SCD, it was stated that the WCD may be considered for adult patients with poor LV systolic function who are at risk of SCD for a limited period, but are not candidates for an ICD (e.g. bridge to transplant, bridge to trans-venous implant, peripartum cardiomyopathy, active myocarditis and arrhythmias in the early phase post-myocardial infarction) [11].

Currently, there is a paucity of data on the long-term outcome of patients after termination of WCD therapy. To our knowledge, this is one of the first studies providing continued clinical and echocardiographic follow-up data after WCD therapy, irrespective of subsequent ICD implantation. Furthermore, we evaluated whether the WCD, as an external monitoring system with an integrated bradycardia alarm, can contribute to the optimization of device selection in patients who need ICD implantation after wearing a WCD.

## Methods

### Patient recruitment

This observational cohort study included all patients receiving a WCD at our tertiary care University Center between 2012 and September 2016. All patients were fitted with a ZOLL Life Vest™ system (Pittsburgh, USA).

All patients received OMT. Each subject provided consent for the de-identified analysis of standard clinical data. The study was approved by the local ethics committee and conforms to the 1975 Declaration of Helsinki.

### The wearable cardioverter-defibrillator

The WCD ZOLL Life Vest™ system (Pittsburgh, USA) has been described previously [8]. The WCD programming was individually adapted to the patient’s underlying heart disease and electrocardiographic patterns. In general, for older patients the ventricular tachycardia (VT) zone was programmed at a heart rate of 150 bpm with a VT response time of 60 s and the ventricular fibrillation (VF) zone at a rate of 200 bpm with a VF response time of 25 s. For younger and more active patients the VT zone was programmed at a heart rate of 180 bpm. First shock energy was set at maximum output (150 J) in all patients. Any arrhythmia episode was considered as a separate episode when occurring with a minimum delay of 3 min from the previous one. Each individual episode was reviewed and classified into the following categories: sustained VT (lasting 30 s or longer) or VF with WCD shock therapy, non-sustained VT (lasting less than 30 s), bradycardia of 30 beats per minute or less or asystole. Inappropriate WCD therapy was classified as a non-VT/− VF episode treated by WCD shock.

### Follow-up and data collection

Data were prospectively collected from the time of the initial hospitalization with WCD implementation. Baseline data included the indication for WCD, co-morbidities, baseline medications, ECG data, and echocardiographic results. LVEF was calculated using Simpsons’s method. WCD was generally prescribed for 3 months irrespective of the underlying WCD indication. Data on arrhythmias during follow-up were prospectively collected clinically and simultaneously retrieved from the ZOLL LifeVest Network™.

In patients with primary preventive indication follow-up visits were scheduled 2 months after diagnosis. If LVEF had increased beyond 35% after 2 months, WCD therapy was terminated early. If LVEF had not increased above the 35% threshold, echocardiography was repeated 1 month later. If LVEF remained below 35% on OMT patients received primary prophylactic ICD implantation.

If a patient with primary preventive WCD indication was incompliant and returned the device earlier than planned while LVEF was still below 35%, the patient received normal clinical and echocardiographic controls. An ICD was implanted if LVEF remained below 35% after 3 months on OMT.

If patients had an appropriate WCD shock, a secondary prophylactic ICD was implanted within the following few days. In patients with an indication for cardiac resynchronization therapy (CRT), a cardiac resynchronization therapy defibrillator (CRT-D) was implanted according to current guidelines [4]. Patients with previously explanted ICDs were followed according to clinical indications.

Patients suspected of or demonstrated to have sinus bradycardia, asystole or intermittent AV-block (Mobitz II) or third-degree AV-block received a 2-chamber ICD. Patients with no suspected or reported bradycardia received an S-ICD or a 1-chamber ICD.

Long-term follow-up was counted from the day on which the patient returned the WCD. During long-term follow-up, all study patients irrespective of the duration of WCD therapy or subsequent ICD implantation received clinical and echocardiographic assessments every 6 months and when clinically indicated.

Heart failure medication consisted of angiotensin converting-enzyme inhibitor/angiotensin receptor blocker (ACE-I/ARB), betablocker and mineralocorticoid receptor antagonist (MRA) according to current heart failure guidelines [4]. Procoralan was prescribed in selected cases. Medication doses were adjusted during each follow-up visit. Since January 2016 selected patients received angiotensin receptor-neprilysin inhibitor instead of ACE-I or ARB. Furthermore, all patients were screened for iron deficiency in heart failure. Intravenous iron substitution was performed if required [4].

For missing data, particularly in cases of missed follow-up visits, the patient or other treating physicians were contacted.

### Statistical analysis

GraphPad PRISM Version 7.0a was used for data analysis. Data are presented as mean ± standard deviation or median (range) for continuous variables or as number of cases for categorical variables. Baseline characteristics were compared by t-test for parametric continuous variables and by Fishers exact test (4 groups) or χ2 test (more than 4 groups) for categorical variables. For analysis of longitudinal evolution and more than 2 groups with paired nonparametric data the Friedman test with Dunn’s multiple comparisons post-test was used. A two-tailed *P* value < 0.050 was considered statistically significant.

## Results

### Patient’s baseline data

Between April 2012 and September 2016 114 patients were prescribed a WCD. Eight patients returned their WCD during the first hours after initiation because of unwillingness or inability to handle it. One patient was lost to follow-up. 105 patients had complete data sets of baseline and follow-up during and after WCD use and were included in the data analysis.

**Fig. 1:**
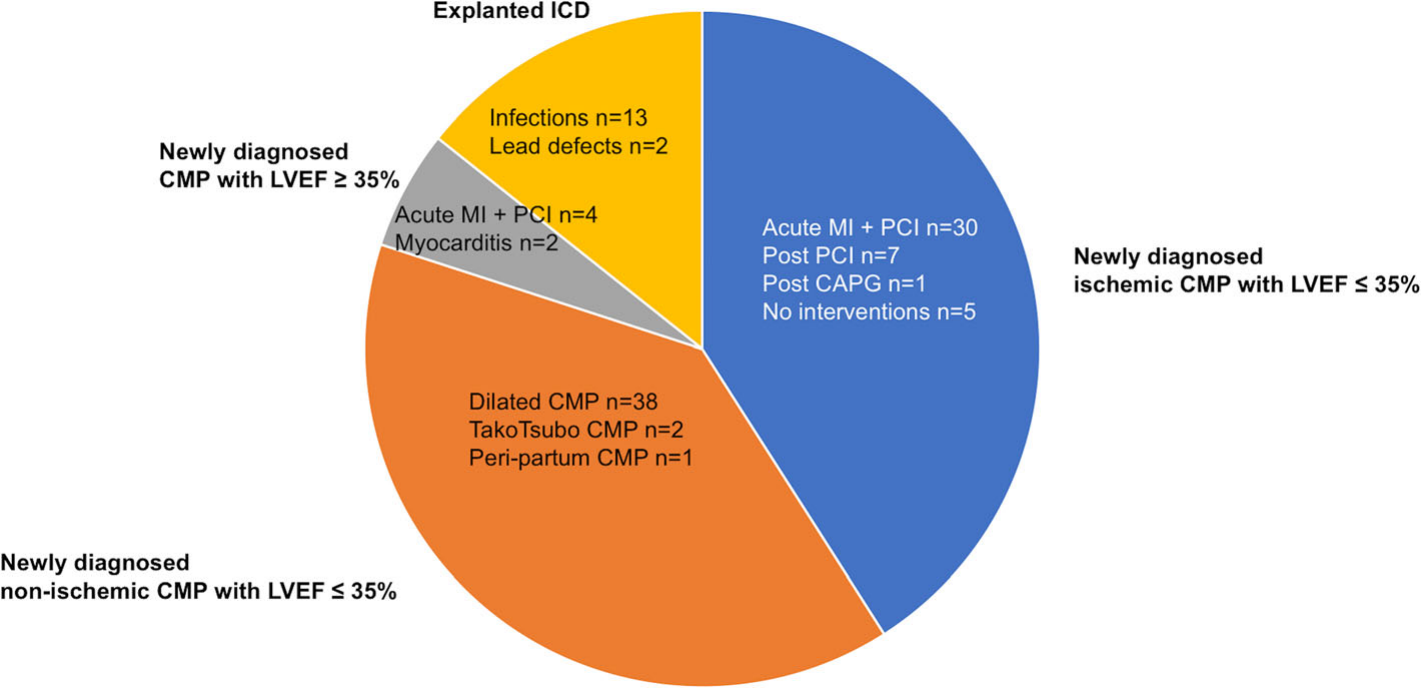
WCD indications of the 105 patients

Patients were classified according to WCD indication: The most common indication for WCD (in 84 of 105 patients) was primary preventive therapy in patients with heart failure symptoms and newly diagnosed ICM or NICM with baseline LVEF ≤35% (Fig. 1, Table 1). These patients received the WCD to bridge the duration of implementing OMT to monitor whether LVEF rose above 35%. Further indications are shown in Fig. 1.

**Table 1:** Patient’s baseline data at prescription of WCD

Total number of patients	All patients	Newly diagnosed ICM	Newly diagnosed NICM	ICD explant	Newly diagnosed CMP
		LVEF ≤ 35%	LVEF ≤ 35%		LVEF ≥ 35%
	*n* = 105	*n* = 43	*n* = 41	*n* = 15	*n* = 6
Age, years, median (range)	60 (26–79)	62 (43–78)	54 (30–78)	62 (29–79)	53 (26–63)
Male sex, n (%) BMI kg/ m^2^, mean ± SD	82 (78.1) 28.6 ± 6.6	35 (81.4) 28.2 ± 6.2	28 (68.3) 29.8 ± 7.1	14 (93.3) 28.7 ± 6.7	5 (83.3) 23.5 ± 3.6
Arrhythmias before WCD					
Atrial flutter or fibrillation, n (%)	34 (32.4)	10 (23.3)	15 (36.6)	8 (53.3)	1 (16.7)
Previous VT/VF/SCD, n (%)	21 (20.0)	6 (14.0)	2 (4.9)	8 (53.3)	5 (83.3)
Conduction Disorders					
LBBB, n (%)	19 (18.1)	3 (7.0)	10 (24.4)	6 (40.0)	0 (0)
Cardiovascular risk factors					
Diabetes, n (%)	29 (27.6)	14 (32.6)	10 (24.4)	4 (26.7)	1 (16.7)
Hypertension, n (%)	71 (67.6)	38 (88.4)	16 (6.6)	13 (86.7)	4 (66.6)
Hyperlipidemia, n (%)	62 (59.0)	31 (72.1)	17 (41.5)	12 (80.0)	2 (33.3)
Concomitant diseases					
Kidney Dysfunction, n (%)	20 (19.0)	10 (23.3)	6 (14.6)	3 (20.0)	1 (16.7)
COPD, n (%)	9 (8.6)	3 (7.0)	3 (7.3)	2 (13.3)	1 (16.7)
NYHA, mean ± SD	2.7 ± 1.2	2.7 ± 1.3	3.0 ± 1.0	2.5 ± 0.9	1.3 ± 0.5
LVEF baseline, mean % ± SD	28.3 ± 9.8	28.9 ± 6.0	23.3 ± 6.9	32.2 ± 13.3	49.0 ± 7.5
Medication					
Betablocker, n (%)	102 (97.1)	41 (95.3)	40 (97.6)	15 (100.0)	6 (100.0)
ACE-I/ARB, n (%)	97 (92.4)	42 (97.7)	39 (95.1)	11 (73.3)	5 (83.3)
MRA, n (%)	67 (63.8)	25 (58.1)	35 (85.4)	5 (33.3)	2 (33.3)
ARNI, n (%)	1 (1.0)	0 (0)	1 (2.4)	0 (0)	0 (0)
Procoralan, n (%)	6 (5.7)	0 (0)	5 (12.2)	1 (6.7)	0 (0)
Diuretic, n (%)	71 (67.6)	26 (60.5)	31 (75.6)	12 (80.0)	2 (33.3)
Amiodarone, n (%)	12 (11.4)	1 (2.3)	5 (12.2)	6 (40.0)	0 (0)

*ACE*-I = angiotensin converting-enzyme inhibitor, *ARB* = Angiotensin receptor blocker, *ARNI* = angiotensin receptor-neprilysin inhibitor, *BMI* = Body mass index*, CABG* = coronary artery bypass graft, *CMP* = cardiomyopathy, *COPD* = chronic obstructive pulmonary disease, *ICD* = implantable cardioverter-defibrillator, *ICM* = ischemic cardiomyopathy, *LBBB* = left bundle branch block, *LVEF* = left ventricular ejection fraction, *MRA* = mineralocorticoid receptor antagonist, *n* = number of patients, *NICM* = non-ischemic cardiomyopathy, *PCI* = percutaneous coronary intervention, *RBBB* = right bundle branch block, *SCD* = sudden cardiac death*, SD* = standard deviation, *VT* = ventricular tachycardia, *VF* = ventricular fibrillation, *WCD* = wearable cardioverter-defibrillator

Median patient age of the whole patient population was 60 years with a male predominance (82%). Further details of the patients’ baseline data are presented in Table 1.

Comparing baseline data of patients with newly diagnosed ICM and NICM, patients with ICM were older (ICM versus NICM, median (range): 62 (43–78) versus 54 (30–78)) (*p* = 0.0008) and had a lower prevalence of left bundle branch block (*p* = 0.03) than patients with NICM. They were more likely to suffer from arterial hypertension (*p* < 0.0001) and hyperlipidemia (*p* = 0.005) than patients with NICM and were less often treated with mineralocorticoid-receptor antagonists (*p* = 0.008). LVEF was significantly higher in ICM patients than in NICM patients (*p* = 0.0002).

### WCD data

The mean WCD wear time of all 105 patients was 68.8 ± 50.4 days with a mean daily use of 21.5 ± 3.5 h (Table 2).

**Table 2:** Follow-up during and after WCD use

Total number of patients	All patients	Newly diagnosed ICM	Newly diagnosed NICM	ICD explant	Newly diagnosed CMP
		LVEF ≤ 35%	LVEF ≤ 35%		LVEF ≥ 35%
	*n* = 105	*n* = 43	*n* = 41	*n* = 15	*n* = 6
Wearing days, mean ± SD	68.8 ± 50.4	57.8 ± 42.6	69.2 ± 39.6	92.5 ± 87.0	70.2 ± 31.2
Wearing hours/day, mean ± SD	21.5 ± 3.5	21.0 ± 3.8	21.3 ± 3.5	23.1 ± 1.1	22.4 ± 1.7
LVEF % baseline, mean ± SD	28.3 ± 9.8	28.9 ± 6.0	23.3 ± 6.9	32.2 ± 13.3	49.0 ± 7.5
LVEF % end of WCD use, mean ± SD	36.1 ± 11.5	36.3 ± 10.3	34.8 ± 11.1	32.9 ± 13.0	50.8 ± 8.6
(*p* value of LVEF baseline versus end of WCD use)	(*p* < 0.001)	(*p* < 0.001)	(*p* < 0.001)	(*p* = n.s.)	(*p* = n.s.)
ICD implanted					
Total n (%)	54 (51.4)	21 (48.8)	18 (43.9)	13 (86.7)	2 (33.3)
Days WCD start to ICD implantation, mean ± SD	103.8 ± 73.7	127.7 ± 84.1	90.0 ± 36.8	94.7 ± 90.3	37.0 ± 18.4
Single-chamber ICD, n (%)	8 (7.6)	3 (7.0)	4 (9.8)	0 (0)	1 (16.7)
Dual-chamber ICD, n (%)	4 (3.8)	1 (2.3)	0 (0)	2 (13.3)	1 (16.7)
CRT-D, n (%)	12 (11.4)	3 (7.0)	5 (12.2)	4 (26.7)	0 (0)
S-ICD, n (%)	30 (28.6)	14 (32.6)	9 (22.0)	7 (46.7)	0 (0)
No ICD implanted					
Total, n (%)	51 (48.6)	22 (51.2)	23 (56.1)	2 (13.3)	4 (66.7)
LVEF recovery to > 35%, n (%)	40 (38.1)	19 (44.2)	21 (51.2)	0 (0)	0 (0)
Transient WCD indication, n (%)	4 (3.8)	0 (0)	0 (0)	0 (0)	4 (33.3)
Patient refused ICD, n (%)	5 (4.8)	2 (4.7)	2 (4.9)	1 (6.7)	0 (0)
Death during WCD wear, n (%)	2 (1.9)	1 (2.3)	0 (0)	1 (6.7)	0 (0)
Arrhythmic death during WCD wear, n (%)	0 (0)	0 (0)	0 (0)	0 (0)	0 (0)

*CRT-D* = cardiac resynchronization therapy with defibrillator*, ICM* = ischemic cardiomyopathy, *ICD* = implantable cardioverter-defibrillator, *LVEF* = left ventricular ejection fraction, *n* = number of patients, *NICM* = non-ischemic cardiomyopathy, *SD* = standard deviation*, S-ICD* = subcutaneous ICD, *WCD* = wearable cardioverter-defibrillator

Of the 84 patients with primary preventive indication, 37 returned their WCD earlier than the prescribed 3 months due to early LVEF improvement (21 of 37 patients), non-compliance (12 of 37 patients), early ICD-implantation due to an appropriate WCD shock (3 of 37 patients) and non-cardiac death (1 of 37 patients).

Mean WCD wear time was 57.8 ± 42.6 days in patients with newly diagnosed ICM with LVEF ≤ 35% and 69.2 ± 39.6 days in patients with newly diagnosed NICM with LVEF ≤ 35% (*p* = n.s.).

Patients with a previous explanted trans-venous ICD had a mean WCD wear time of 92.5 ± 87.0 days.

Patients with previous explanted ICDs had a higher mean daily use (23.1 ± 1.1 h/day) than did patients in the other patient groups (Table 2). This was caused by the higher risk awareness in this patient group.

Five of the 105 patients (4.8%) received appropriate WCD shocks. Characteristics of these patients are presented in Table 3. Four patients had WCD shock because of VF, one patient experienced WCD shock because of VT. In all five patients VT or VF was successfully terminated with the first WCD shock. In each of the five patients WCD shocks occurred after discharge from hospital between 3 to 154 days after initiation of WCD.

**Table 3:** Characteristics of patients with appropriate WCD shocks

Patient, Age	Indication for WCD	Baseline LVEF	Shock days from start of WCD	Type of arrhythmia	1.Shock successful	Time to shock
Male, 64	ICM STEMI, PCI RCA	35%	72	VF	yes	54 s
Male, 50	ICM STEMI, PCI LAD	25%	3	VF	yes	46 s
Male, 45	Dilated CMP Previous explant of primary preventive trans-venous ICD	30%	67	VF	yes	49 s
Male, 71	ICM WCD because of systemic infection that precluded ICD implant	20%	154	VT with heart rate 180/min	yes	69 s
Female, 26	Myocarditis	53%	38	VF	yes	63 s

*WCD* = wearable cardioverter-defibrillator, *PCI* = percutaneous coronary intervention, *VT* = ventricular tachycardia, *VF* = ventricular fibrillation, *LVEF* = left ventricular ejection fraction, *ICM* = ischemic cardiomyopathy, *sec* = seconds

The single patient who received an appropriate WCD shock after 154 days of WCD wear was a 71-year-old man with ICM. He received prolonged WCD therapy due to persistent bacterial infection triggered by his knee prosthesis. The WCD shock was delivered because of sustained VT with ventricular heart rate of 180/min. Then, an S-ICD was successfully implanted within the following days.

One patient had asymptomatic non-sustained VT that was detected via the ZOLL LifeVest Network™. Furthermore, one patient had an asystole of 10s during sleep that was detected via the ZOLL LifeVest Network™.

One 74-year-old female patient with mild cognitive defects and newly diagnosed ICM received an inappropriate WCD shock on day 3. This was triggered by artifactual voltage fluctuations misinterpreted by the WCD as ventricular arrhythmia. The patient had ignored both tactile and audible alarms and failed to press the response button of her WCD.

None of the 21 patients with early LVEF improvement and none of the 12 patients who returned their WCD early due to non-compliance showed signs of ventricular tachyarrhythmias during the missed WCD time period.

### Changes in LVEF and ICD implantations after WCD wear

#### LVEF assessment

At the end of WCD wear mean LVEF of all 105 patients had improved from 28.3 ± 9.8% at baseline to 36.1 ± 11.5% (*p* < 0.001) (Table 2).

**Fig. 2:**
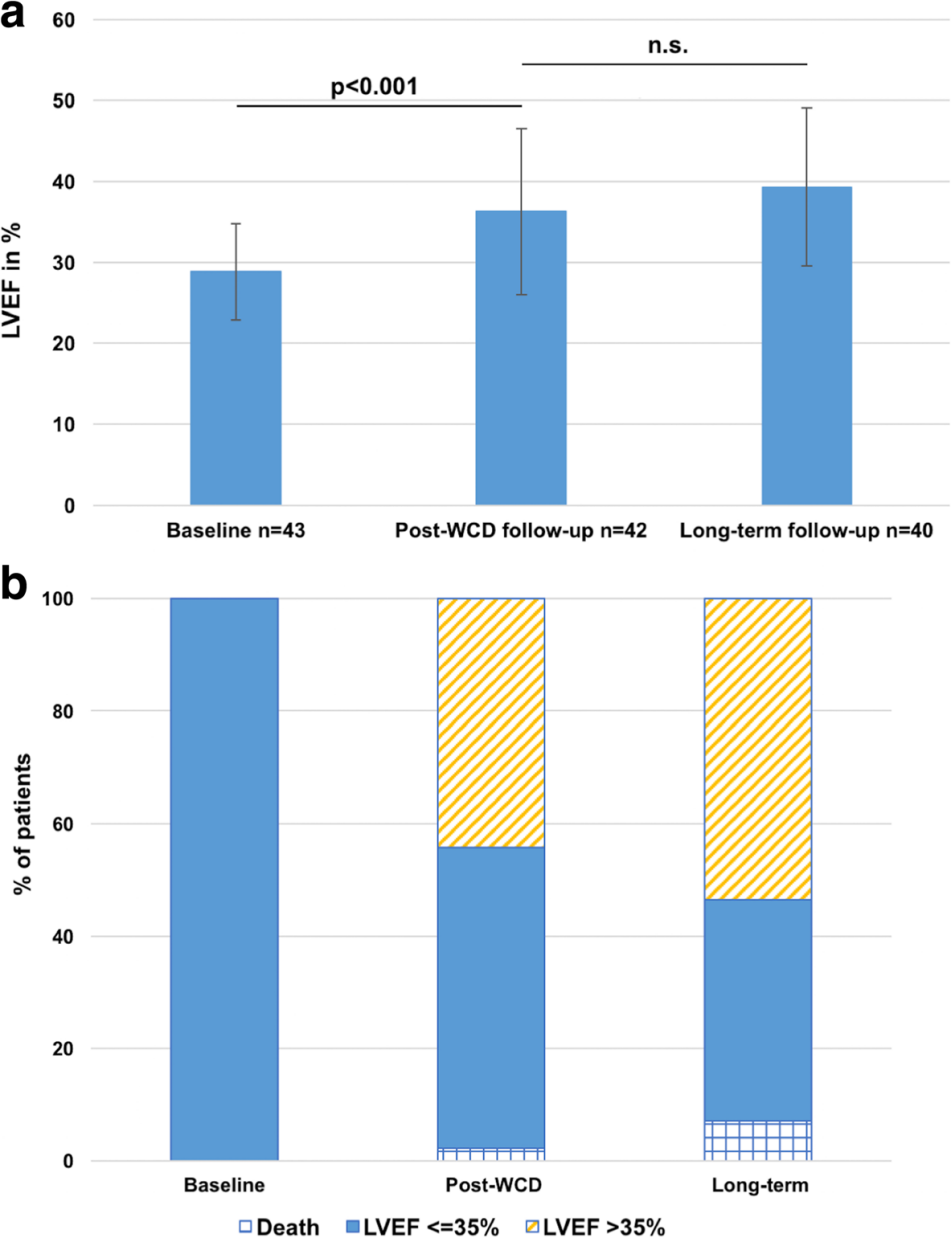
**a** Evolution of LVEF during follow-up in patients with newly diagnosed ICM with LVEF ≤35% (mean ± SD). **b** Evolution of LVEF/ICD indication in patients with newly diagnosed ICM with LVEF ≤ 35%

The 43 patients with newly diagnosed ICM with LVEF ≤35% showed a significant improvement of mean LVEF from 28.9 ± 6.0% to 36.3 ± 10.3% (*p* < 0.001) (Fig. 2a). 19 of the 43 patients (44.2%) showed improvement of LVEF beyond 35% and surpassed the threshold for ICD implantation (Fig. 2b). 54.8% of the patients alive had persistent primary prophylactic ICD indication.

**Fig. 3:**
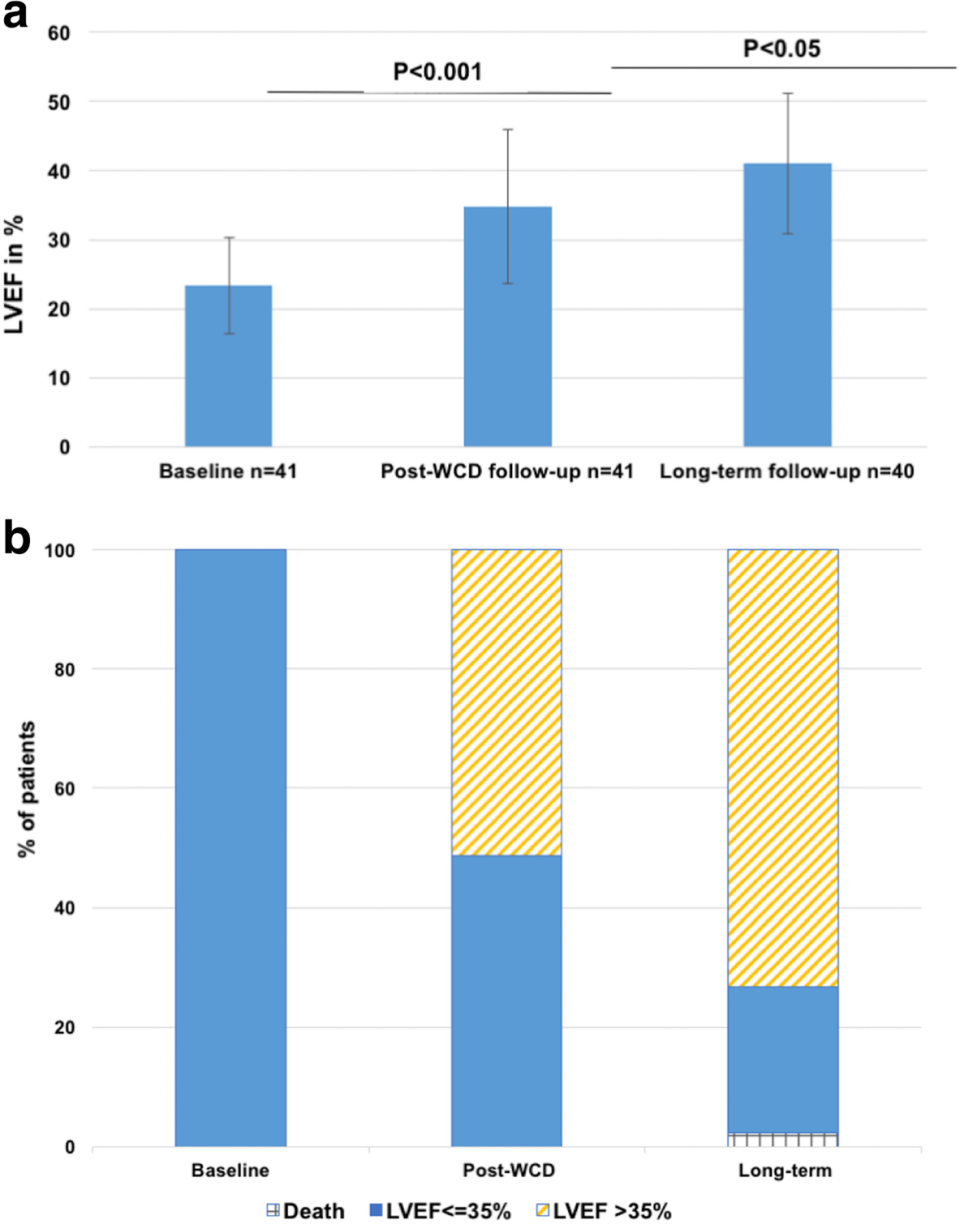
**a** Evolution of LVEF during follow-up in patients with newly diagnosed NICM with LVEF ≤35% (mean ± SD). **b** Evolution of LVEF/ICD indication in patients with newly diagnosed NICM with LVEF ≤ 35%

The 41 patients with newly diagnosed NICM with LVEF ≤35% also showed significant improvement of mean LVEF from 23.3 ± 6.9% to 34.8 ± 11.1% (*p* < 0.001) (Fig. 3a). 21 of the 41 patients (51.2%) showed improvement of LVEF beyond 35% (Fig. 3b). 48.8% had persistent primary prophylactic ICD indication.

Patients with explanted ICDs showed no significant changes in LVEF (32.2 ± 13.3% at baseline to 32.9 ± 13.0% at the end of WCD wear) (*p* = n.s.). Furthermore, patients with newly diagnosed CMP and LVEF ≥ 35% had no significant changes in LVEF (49.0 ± 7.5% at baseline to 50.8 ± 8.6% at the end of WCD wear) (*p* = n.s.).

#### ICD implantations

An ICD was implanted in 54 (51.4%) out of 105 patients (Table 2). Mean time to ICD implantation was 103.8 ± 73.7 days from the start of WCD wear. In the patient group with newly diagnosed ICM 21 patients (48.8%) received an ICD after 127.7 ± 84.1 days from starting WCD. In the patient group with newly diagnosed NICM 18 patients (43.9%) received an ICD after 90 ± 36.8 days from starting WCD. ICD implantation rate was highest in patients with a previous explanted ICD (86.7%).

The five patients with appropriate WCD shocks all received secondary prophylactic ICD. The patient that had asystole detected by the WCD received a 2-chamber ICD.

**Fig. 4:**
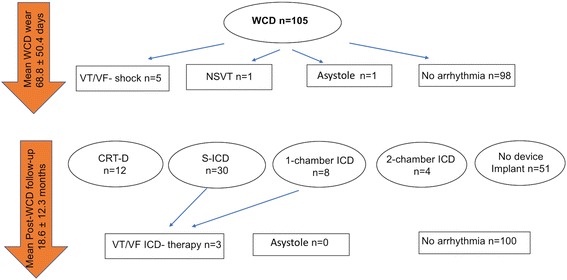
Overview arrhythmias and devices

Figure 4 provides an overview of the patients’ arrhythmias and distribution of implanted devices. In total, eight of the 54 patients with ICD received a single-chamber ICD, 4 patients received a dual-chamber ICD, and 12 patients received a CRT-D. Furthermore, 30 patients without prior evidence of bradyarrhythmias received a subcutaneous ICD (S-ICD).

Fifty-one of the 105 patients (48.6%) did not receive an ICD (Table 2): 40 patients with ICM/ NICM and LVEF < 35% had LVEF improvement beyond 35%, five patients refused recommended ICD implantation and in four patients with newly diagnosed CMP and LVEF > 35% WCD indication was only transient. Two patients had died while wearing the WCD. The cause of death was endocarditis followed by septic shock in one patient and pulseless electrical activity and pumping failure after hemodialysis in the other. Neither clinical observation nor the ZOLL LifeVest Network™ showed evidence of tachyarrhythmia or bradyarrhythmia in either patient.

### Long-term follow-up after discontinuation of WCD

After end of WCD use all 103 patients alive were followed for a mean period of 18.6 ± 12.3 months- irrespective if they had received an ICD or not.

#### LVEF during long-term follow-up

Mean LVEF of all patients further improved from 36.1 ± 11.5% at end of WCD wear to 40.7 ± 11.0% at last follow-up (*p* < 0.001).

In the patient group with newly diagnosed ICM mean LVEF changed not significantly from 36.3 ± 10.3% at end of WCD wear to 39.3 ± 9.8% at last follow-up (*p* = n.s.; Fig. 2a). Mean change of LVEF from baseline to last follow-up was 9.4 ± 1.8%. 4 of the 43 patients (9.3%) with newly diagnosed ICM showed late LVEF recovery to > 35%. During long-term follow-up, the proportion of patients with LVEF beyond 35% increased not significantly from 44.2% at end of WCD wear to 53.5% (p = n.s.; Fig. 2b). At last follow-up 42.5% of the patients alive had persistent LVEF ≤ 35%.

In the patient group with newly diagnosed NICM mean LVEF significantly improved from 34.8 ± 11.1% at end of WCD wear to 41.0 ± 10.2% at last follow-up (*p* < 0.05; Fig. 3a). Mean change of LVEF from baseline to last follow-up was 17.7 ± 1.9%. Nine patients (22.0%) showed late recovery of LVEF to > 35%. The proportion of patients with LVEF ≥35% significantly increased from 51.2% at end of WCD wear to 73.2% (*p* = 0.038; Fig. 3b). At last follow-up only 25.0% of the patients alive had LVEF ≤35%.

#### Arrhythmias during long-term follow-up

During long-term follow-up three of the 54 patients (5.6%) with implanted ICDs received a total of 16 appropriate ICD treatments (Table 4). One patient received one S-ICD shock because of VT. Another patient received 13 ATPs from his single-chamber ICD because of a recurrent monomorphic VT that was subsequently ablated. Both of them had ICM with LVEF < 35% that did not improve throughout the study.

**Table 4:** Long-term follow-up after discontinuation of WCD

	ICD implanted	No ICD implanted
	*n* = 54	*n* = 49
Sustained VT/VF, n (%)	3 (5.6)	0 (0)
Appropriate ICD therapy, n (%)	3 (5.6)	–
Inappropriate ICD therapy, n (%)	2 (3.7)	–
Asystole, n (%)	0 (0)	0 (0)
Death, n (%)	1 (1.9)	2 (4.1)
Arrhythmic death, n (%)	0 (0)	0 (0)
Non-arrhythmic death, n (%)	1 (1.9)	2 (4.1)

*ICD* = implantable cardioverter-defibrillator, *n* = number of patients, *VT* = ventricular tachycardia, *VF* = ventricular fibrillation, *WCD* = wearable cardioverter-defibrillator

Another patient who had already received an appropriate WCD shock for VF received two appropriate shocks of his single-chamber ICD for VF. He was subsequently treated with amiodarone. His WCD indication was newly diagnosed ICM and baseline LVEF < 35%. His LVEF did not change during WCD wear but improved during long-term follow-up and the appropriate ICD shocks occurred while LVEF was 42%.

None of the patients that had no ventricular tachyarrhythmias during WCD wear and who showed late LVEF improvement beyond 35% had ventricular tachyarrhythmias throughout the study.

None of the 30 patients implanted with an S-ICD showed evidence of bradycardia and none of these patients needed subsequent implantation of a pacemaker or device change to a trans-venous ICD system.

Furthermore, none of the 51 patients who had not received an ICD showed evidence of ventricular tachyarrhythmias.

#### Inappropriate shocks during long-term follow-up

Two out of the 54 patients (3.7%) with ICD had inappropriate shocks (Table 4): one patient had one inappropriate shock within hours after S-ICD implantation that was caused by residual air in the vicinity of the newly implanted sensing electrode which led to a decrease of the S-ICD signal amplitude and abrupt baseline shift. The trapped air dissolved spontaneously and no further sensing abnormalities were observed during follow-up. Furthermore, one patient who was implanted with a single-chamber ICD had one inappropriate shock because of atrial fibrillation.

#### Deaths during long-term follow-up

Three patients died during long-term follow-up (Table 4), and all three died because of non-arrhythmic reasons. Two patients without permanent ICD died because of septic shock. One patient who had received an S-ICD died of lung empyema 14 months later. Postmortem ICD interrogation showed no evidence of ventricular arrhythmia. No patient died from SCD.

## Discussion

The major findings of our study were as follows

1. WCD therapy proved to be safe and effective. All patients, independent of WCD indication, were successfully bridged to either LVEF recovery or ICD implantation.
2. Both patient groups with newly diagnosed ICM and with NICM showed significant LVEF improvement during WCD wear. Following WCD treatment, ICD implantation could be avoided in almost half of the patients. During long-term follow-up, further significant LVEF improvement was observed in patients with NICM.
3. The WCD, as an external monitoring system for detecting asystole or bradycardia, contributed important information for device selection in patients who needed ICD implantation after WCD wear. During long-term follow-up, no patient with S-ICD needed change to a trans-venous device. Furthermore, none of the patients without an implanted ICD showed any signs of ventricular tachyarrhythmias.

### WCD efficacy and safety

Several observational studies have shown WCD to successfully identify and interrupt VT and VF with shock efficacy rates of 99–100% [8–10, 12]. The present study confirmed effectiveness and safety of the WCD therapy. 4.8% of the patients had sustained VT/VF, and all episodes were successfully terminated by WCD shock.

The percentage of VT/VF was slightly higher than in multi-center registries reporting appropriate WCD shocks in 1.1 to 2.1% of the patients [8–10]. Other single center data showed similar results to our patients with appropriate shocks in 3.9 to 7% of patients [13–16].

Several publications have reported higher WCD shock rates in patients with newly diagnosed ICM than in patients with newly diagnosed NICM [9, 16, 17]. Sing et al. even questioned the utility of the WCD in patients with NICM [17]. Nevertheless, Duncker et al. found an incidence of VT in the early stage of NICM of 38.7 of 100 person-years, reflecting the relevant arrhythmogenic risk in patients with NICM and non-optimized heart failure medication [18]. High rates of appropriate WCD shocks have been described in several publications evaluating patients with explanted ICDs [8, 10]. As a result, WCD prescription is now generally accepted in this patient group [11].

In our study, the majority of rescued patients had new onset ICM. However, one patient with NICM and LVEF < 35% whose primary prophylactic ICD had to be explanted and another patient with myocarditis also received lifesaving WCD shocks because of VF. The numbers of patients in the four groups in our study (especially in the patient group with explanted ICDs and myocarditis) were too small to permit interesting conclusions about predictive factors for arrhythmias. Nevertheless, our data confirm the usefulness of WCD in both patients with ICM and NICM and support its application in patients with a wide range of indications.

One of the problems associated with WCD therapy is incorrect use [19] which caused inappropriate shock in one patient. However, rates of inappropriate WCD shocks were low (0.4–3.0%) [8–10, 12]. Importantly, no death has been attributed to WCD technical failure since its introduction [20].

Another common problem [19] we faced in the study was that several patients were reluctant to wear the WCD or returned the device earlier than planned. Poor compliance or inappropriate use of the WCD may have catastrophic consequences [19]. Patient education on how to properly wear the device, change the battery and disable shock delivery is crucial. Furthermore, patients should understand their cardiac disease and the potential benefits associated with the use of the WCD. Patients should be selected carefully and the device should not be deployed in patients unfit or unwilling to properly manage it. Because WCD use may be monitored online, detection of incorrect use of the device is possible and should be used to provide prompt feedback and motivation to a non-compliant patient. In non-compliant patients who are at high risk for ventricular tachyarrhythmias, other forms of monitoring or therapy should be discussed (patient monitoring in-hospital, early ICD implantation, etc.)

### LVEF improvement

ICD implantation rate in the whole patient cohort was 54%. This parallels other studies with similar patient collectives that reported implantation rates of 34 to 57% [9, 14, 16, 21, 22]. Naturally, ICD implantation rate was highest in the patient group with a prior explanted trans-venous ICD. Following WCD, ICD implantation could be avoided in almost half of the patients with ICM and NICM. LVEF improvement was the most common reason not to implant an ICD.

Current heart failure guidelines recommend primary preventive ICD implantation in both patients with ICM and NICM with LVEF ≤35% despite at least 3 months of OMT [4]. OMT is currently defined as combination of high dose betablocker and high dose angiotensin converting-enzyme inhibitor, with the addition of a mineralocorticoid receptor antagonist in patients with persistent symptoms of heart failure [4]. As a result, ICD implantation 3 months after diagnosis might be too early in many patients because patients would be assumed to be on OMT already at the moment of diagnosis.

A previous study including patients with acute myocardial infarction who were treated with modern therapies including early revascularization, showed greatest LVEF improvement within the first month. Nevertheless, a smaller part of patients showed LVEF improvement beyond the initial 40 days after acute myocardial infarction [23].

On the other hand, recent studies including patients with NICM reported late LVEF improvement after implantation of a primary preventive ICD [24, 25]. Verma et al. reported LVEF improvement to > 35% in 12% of patients [24]. Grimm et al. found LVEF improvement in 24% of patients with NICM after ICD implantation reducing incidence of appropriate ICD therapies to ∼1% per year in patients with improved LVEF [25]. In both studies, multivariate analysis identified no other significant predictor for LVEF improvement after ICD implantation than a short time from diagnosis to device implant. Consistent with the data of Varma and Grimm, 22.0% of our patients with NICM showed late LVEF improvement beyond 35%. It should be noted that approximately half of the NICM patients in our study who had late LVEF improvement had received a CRT-D device (12.2%). The CRT is known to have an important effect on LVEF recovery.

In patients with ICM we observed a trend towards late LVEF improvement. 9.3% of ICM patients skipped the ICD threshold during long-term follow-up and developed LVEF > 35% while 7.0% had received CRT-D.

Patients in our study received optimization of medical therapy not only during WCD period but were seen every 6 months in our heart failure outpatient clinic where doses of medication were adjusted. Our study overlapped the debut of newer heart failure therapies such as sacubitril/valsartan which may have played a role in improving LVEF. Sacubitril/valsartan has been shown to improve morbidity and mortality in heart failure [1]. Furthermore, this medication has also been shown to reduce the incidence of VT and arrhythmogenic deaths which might contribute to the low rate of shocks delivered to patients who received an ICD [26].

A recently published study by Duncker et al. which included 156 patients with newly diagnosed NICM or ICM (with a majority of NICM) showed 11 ± 11% improvement in LVEF during 3 months of OMT and WCD use [15]. Even in patients without CRT-D implant they demonstrated improvement of LVEF to > 35% in 33% of their patients during a prolonged period of OMT beyond 3 months [15]. Nevertheless, patients had a relevant risk for life-threatening VT as long as LVEF was ≤35%. Therefore, they proposed a prolonged regimen of WCD use beyond 3 months in patients with one of the three indications: improvement of LVEF to 30–35% or Δ LVEF ≥5% during the first 3 months of WCD wear or insufficient optimization of medical dosages (especially MRA).

Duncker et al. did not stratify subjects by etiology of heart failure (ICM vs NICM) which might have improved their ability to personalize indications for WCD use. Conversely the smaller numbers of subjects in the present study preclude further subanalysis of specific patient subgroups who might benefit from prolonged use of WCD or ICD implantation reduction.

The recently published DANISH trial confirmed the risk of life-threatening ventricular arrhythmias in the chronic phase of NICM, during which primary preventive ICD implantation did not reduce overall mortality [27]. Given that primary preventive ICD implantation in NICM has become debatable, a prolonged WCD regimen might offer prevention from SCD during careful optimization of heart failure therapy and may avoid too early or even not mortality reducing ICD implantation in patients with NICM.

In general, the potential for LVEF improvement indicates a more favorable outcome in both patients with ICM and NICM [28]. Nevertheless, existing guidelines, especially for patients with dilated cardiomyopathy, lack sensitivity and specificity in the selection of patients who need primary- prevention ICD implantation. There is a need for a more precise risk stratification algorithm that considers new markers other than LVEF to provide a more comprehensive system for disease phenotyping. Such new promising markers include the extent or pattern of myocardial replacement fibrosis detected by late gadolinium enhancement cardiac magnetic resonance imaging, cardiac autonomic dysfunction detected by ^123^I-meta-iodobenzylguanidine myocardial scintigraphy, several ECG-derived markers and genetic testing [29, 30]. Future ICD studies should also consider the antiarrhythmic effects of modern heart failure therapies such as sacubitril/valsartan.

### ICD implantations

Next to its function to detect and terminate VT/VF the WCD also acts as an external monitoring system to identify bradycardias and asystole. Asymptomatic asystole was identified in one patient who subsequently received a 2-chamber ICD. Currently the WCD only records bradycardias with heart rates less than 10/min. Recording of less severe bradycardia might help to better identify patients at risk for symptomatic bradycardia.

In our study 30 patients were transitioned to S-ICD after wearing the WCD. The S-ICD, like the WCD, is an innovative device without the need to implant trans-venous ICD leads avoiding lead related complications. The S-ICD, similar to the WCD, has no permanent pacing functions and is therefore not indicated in patients who need bradycardia support, cardiac resynchronization or antitachycardiac pacing [11]. During post WCD long-term follow-up, both trans-venous ICDs and S-ICDs successfully terminated VT/VF. Importantly, none of the S-ICD patients needed implantation of a pacemaker or device change to a trans-venous ICD system. In the literature, changes from S-ICD to trans-venous ICD due to the need for ATP have been described in about 0.5% to 0.8% of patients [31, 32]. Changes from S-ICD to resynchronization therapy have been described in 0.4% of patients [32]. Furthermore, several cases have been described where a pacemaker was added to S-ICD for ventricular pacing [33, 34]. In our patients, the monitoring function of the WCD helped to optimize patient selection for S-ICD implantation.

### Deaths

Five patients died, primarily because of fatal infection, and no patient died from SCD during the study period. Four of the five patients treated with the WCD had VF as a first arrhythmic event and would likely have died without the WCD therapy. Patients who had received appropriate WCD shocks and patients whose LVEF did not improve beyond 35% were implanted with a permanent ICD. In the other patients, the vulnerable phase until recovery of LVEF was safely bridged with the WCD. None of the patients with recovered LVEF and no ventricular tachyarrhythmia event during WCD wear showed late signs of tachyarrhythmia. Therefore, with the use of the WCD all study patients could be provided a comprehensive safety net avoiding SCD.

#### Limitations of the study

This was a single center study including a variety of patient conditions. It was non-randomized but selection bias was minimized by evaluating all eligible subjects prospectively. Moreover, patients presenting in our department were consecutively included representing a real-world heart failure population. Furthermore, the decision if a 1-chamber ICD or an S-ICD was implanted was made by the operator and not in a randomized manner. It has to be emphasized that the WCD diagnoses bradycardia/ asystole only during wear time. As highgrade AV block was described to occur in about 1% to 2% of heart failure patients per year future need for cardiac pacing cannot be excluded.

## Conclusions

The WCD was confirmed to be safe and effective in protecting patients against arrhythmic death. Patients were successfully bridged to either LVEF recovery or ICD implantation. Future studies are warranted to examine if a prolonged WCD regimen in certain patient groups can minimize risks and costs of permanent ICD implantation while allowing left ventricular reverse remodeling during intensified drug therapy.

It was shown for the first time that the use of the WCD as an external monitoring system can provide important information for an easier decision regarding which kind of ICD should be used for device treatment.

## Abbreviations

ACE-I: Angiotensin converting-enzyme inhibitor
ARB: Angiotensin receptor blocker
ARNI: Angiotensin receptor-neprilysin inhibitor
BMI: Body mass index
CABG: Coronary artery bypass graft
CMP: cardiomyopathy
COPD: Chronic obstructive pulmonary disease
CRT: Cardiac resynchronization therapy
ICD: Implantable cardioverter-defibrillator
ICM: Ischemic cardiomyopathy
J: Joule
LBBB: Left bundle branch block
LVEF: Left ventricular ejection fraction
MI: Myocardial infarction
MRA: Mineralocorticoid receptor antagonist
NICM: Non-ischemic cardiomyopathy
OMT: Optimal medical treatment
PCI: Percutaneous coronary intervention
RBBB: Right bundle branch block
SCD: Sudden cardiac death
SD: Standard deviation
sec: Seconds
S-ICD: Subcutaneous implantable cardioverter-defibrillator
VT: Ventricular tachycardia
WCD: Wearable cardioverter-defibrillator
